# Yield benefits of additional pollination to faba bean vary with cultivar, scale, yield parameter and experimental method

**DOI:** 10.1038/s41598-020-58518-1

**Published:** 2020-02-07

**Authors:** J. Bishop, M. P. D. Garratt, T. D. Breeze

**Affiliations:** School of Agriculture, Policy and Development, University of Reading, Reading, Berkshire RG6 6AR UK

**Keywords:** Agroecology, Ecosystem services, Plant breeding, Plant physiology, Plant reproduction

## Abstract

The benefits of insect pollination to crop yield are used to justify management decisions across agricultural landscapes but current methods for assessing these benefits may underestimate the importance of context. We quantify how the effects of simulated insect pollination vary between five faba bean cultivars, and to what extent this changes between years, scales, yield parameters, and experimental methods. We do this by measuring responses to standardised hand pollination treatments in controlled experiments in flight cages and in the field. Pollination treatments generally improved yield, but in some cases yield was lower with additional pollination. Pollination dependence varied with cultivar, ranging from 58% (loss in yield mass per plant without pollination) in one cultivar, to a lower yield with pollination in another (−51%). Pollination dependence also varied between flight cage and field experiments (−10 to 37% in the same cultivar and year), year (4 to 33%; same cultivar and yield parameter), and yield parameter (−4 to 46%; same cultivar and year). This variability highlights that to be robust, assessments of pollination benefits need to focus upon marketable crop outputs at a whole-plant or larger scale while including and accounting for the effects of different years, sites, methodologies and cultivars.

## Introduction

The contribution of insect pollinators to crop yield has been used to support conservation measures^1^, provide cost-effective agronomic advice^2^, and to support agricultural policies at a national level^3^. Globally, benefits of insect pollination to food production are estimated to be worth $235–577 billion per year^1^. This value is based upon estimates of the “pollinator dependence” of each crop; the proportion of harvestable yield that depends on animal mediated pollination, or how much yield is lost in absence of such pollination.

Recent studies have begun to show that the pollinator dependence of a crop is not fixed and that it interacts with other biological factors (soil quality and predation by pests^4–6^) or agronomic inputs (fertilizer, agrochemicals and water^7–11^), see review^12^.

Pollinator dependence can also vary with crop cultivar, for example in apples^13^ and oilseed rape^14^. Understanding pollinator dependence on a per-cultivar basis could enable crop producers to secure production as pollination services become less predictable^15,16^ following shifts in pollinator populations through time^17,18^. In the short term, producers in landscapes with low levels of semi-natural habitat and pollination service capacity^19^ may benefit from use of low-dependent cultivars, though this needs to be considered alongside other agronomic attributes (e.g. yield potential, disease resistance).

Quantifying variation in pollinator dependence between cultivars has rarely been the primary aim of experiments. Without standardised experimentation it is difficult to identify those cultivars with high or low pollination dependence; as above, each study that estimates the yield benefits of pollination does so with a specific combination of biological and agronomic factors (e.g. climate and soil conditions), complicating between-study comparisons^20^. Studies may also measure yield in different ways and at different scales^21^. For instance the effect of pollination on faba bean (*Vicia faba*) yield has been measured in crop fields^22^, on individual plants^23^, or individual flowers on a plant^24^ but there is currently a lack of information around how well measurements at these different scales are correlated. Without cultivar-specific and context-specific information, it is difficult for producers to consider insect pollination as an agronomic factor^11^ and quantify the benefits of management choices such as creating wildflower and nesting habitats for wild pollinators, using managed pollinators, or selecting particular cultivars.

Faba bean is a grain legume that is often grown as a break crop in arable rotations^25^. The crop is used both for human consumption, and livestock or fish feed^26^. Faba bean is considered to have a mixed-mating breeding system and the pollination dependence of faba bean yield has been shown to vary with weather (seeds per plant 44 to 69%^27^), soil type (pod number 0 to 41% dependent^28^) and short-term heat stress (seed number 12 to 27% dependent^29^). Alongside these factors, it is clear that plant genotype can play an important role in determining pollination dependence of faba bean. Suso & del Río^30^ tested responses of six faba bean genepools to open insect pollination vs exclusion and recorded changes in seed production with insect pollination ranging between a 32% loss to a 37% gain.

In this study, we use three controlled experiments across two years to (i) investigate how pollination dependence varies between cultivars, experimental methods, and years, (ii) examine relationships between yield parameters at different scales and quantify how these relationships are affected by pollination treatments and contextual factors, and (iii) understand how in combination these factors contribute to uncertainty in the economic assessment of pollination benefit.

## Results

### Cage experiment

Cage experiments over two years identified significant differences in the pollination dependency of the five faba bean cultivars tested. There were no differences between the effect of tripping and cross pollination treatments in any cultivar or yield parameter measured (no significant posthoc contrast between any tripping and crossing treatments on the same cultivar) so analyses were conducted with these treatments grouped with no reduction in explanatory power (comparison between ANOVA testing bean number and 2 way interaction of cultivar and pollination with either 3 or 2 levels, F = 0.726, p = 0.699).

**Figure 1:**
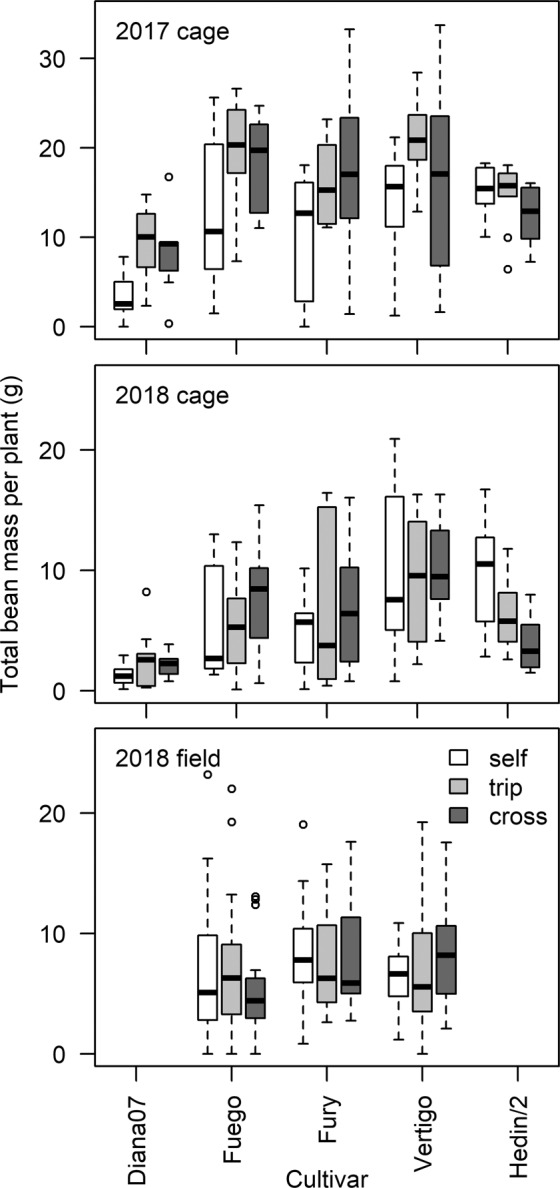
Effect of cultivar, pollination treatment and experimental method on yield mass per plant.

The effect of pollination treatment varied significantly with cultivar for total bean number per plant (interaction between cultivar and pollination treatment; F = 11.4, p < 0.001), total bean mass per plant (F = 4.561, p = 0.001; Fig. 1), total pod number per plant (F = 9.384, p < 0.001) and average number of beans per pod (F = 2.68, p = 0.032), see Table 1. Diana07 and Fuego produced a greater number of beans when a pollination treatment was applied (posthoc tests between selfing and pollination treatments for bean number; p < 0.05) while Hedin/2 produced fewer beans following the pollination treatments (p = 0.006). No cultivars, when taken independently of each other (within cultivar post-hoc contrasts), showed a significant difference between the selfing and pollination treatments for bean mass (though Diana07 was close to significant, p = 0.056). Effects of pollination treatments varied between cultivars depending on the yield parameter in question; for number of beans per pod there was no difference between selfing and additional pollination treatments for Hedin (p = 0.999), Vertigo (p = 0.875), or Diana07 (p = 0.069) while beans per pod increased with pollination by an average of 0.9 in Fury (p = 0.003) and 0.6 in Fuego (p = 0.033). Results were highly variable within individual combinations of pollination treatment and cultivar, see Supplementary Material for extended results table.

The relative pollination dependence of cultivars was consistent across years (3-way interaction of pollination treatment, cultivar and year was not significant for bean number; F = 0.8, p = 0.53, bean mass; F = 0.18, p = 0.95, pod number (F = 1.0, p = 0.4), and beans per pod (F = 1.1, p = 0.4). There was however a large difference in the absolute yield produced between years (c. 27 fewer beans per plant on average in 2018) which resulted in different effects of pollination treatment on yield between the two years (interaction treatment and year for bean number; F = 17.8, p < 0.001, bean mass; F = 5.7, p = 0.017, pod number; F = 8.3, p = 0.004; not significant for beans per pod; F = 1.2, p = 0.28) and different effects of cultivar on yield between the two years (interaction cultivar and year for bean number, F = 6.2, p < 0.001, not significant for bean mass, F = 1.3, p = 0.3, pod number, F = 1.8, p = 0.1; or beans per pod F = 1.0, p = 0.4).

**Figure 2:**
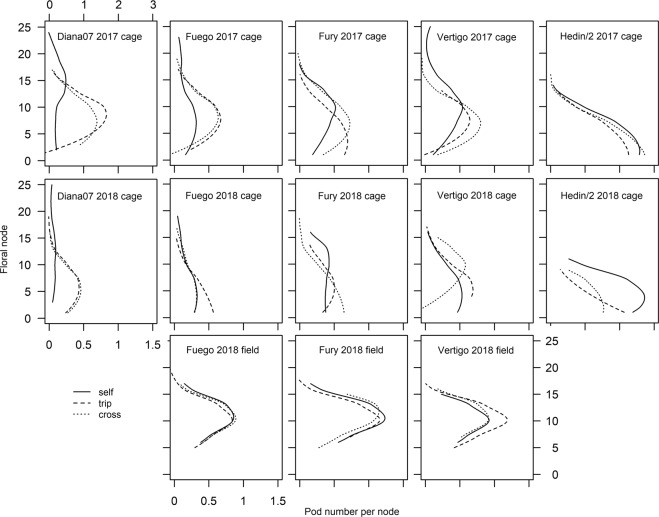
Distribution of pod number per floral node for each cultivar and pollination treatment. Lines are estimated using local polynomial regressions across all replicate plants.

The dominant floral node, around which pods were grouped on the stem, was quantified for each experimental plant in the cage experiments (Fig. 2). There was no difference between the two pollination treatments of tripping and hand- pollination, indicating that cross pollination does not change the distribution of pods on the stem compared to tripping (model including 2 vs 3 factor levels for pollination treatment; p = 0.99). Diana07 showed a significant difference in dominant node position between pollination treatments while other cultivars did not (interaction pollination treatment and cultivar; F = 4.841, p < 0.001). The location of the dominant node was also different between years (1 node lower in 2017; year; F = 4.023, p = 0.046) but relationships between pollination treatments and cultivars did not vary between years (3-way interaction of year, cultivar and pollination treatment; F = 1.532, p = 0.19).

**Figure 3:**
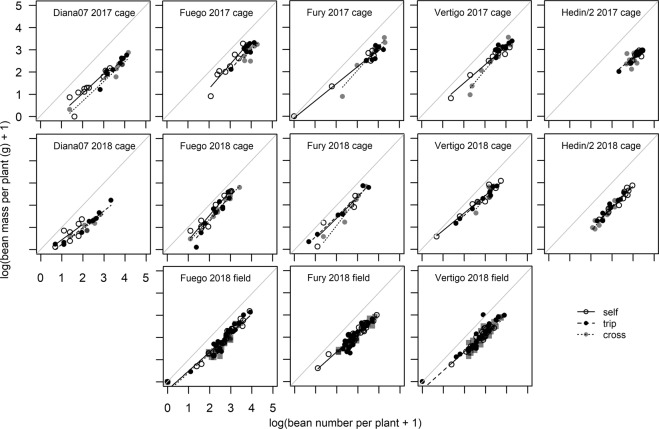
Relationships of total bean number and total bean mass per plant. Lines show model predictions for different pollination treatments and points show data; cross treatment in field experiment is uncaged plants. Diagonal gray lines show 1:1 relationship.

There was a non-linear relationship between total bean number and total bean mass per plant (a model including log(bean number +1) and log(bean mass +1) had the best fit) indicating that mass per bean became progressively lower in plants with more beans (Fig. 3). The different cultivars and the three pollination treatments altered this relationship (deletion test for interaction of bean number, pollination treatment and cultivar; F = 3.067, p = 0.003; comparison between model including pollination with either 3 or 2 levels, F = 2.744, p = 0.002). Fury (p = 0.023) and Vertigo (p = 0.024) plants receiving hand pollination maintained significantly more mass as their number of beans increased compared to Diana07 plants (Fig. 3) indicating that Fury and Vertigo were less affected by resource limitation than Diana07.

### Field experiment

Pollination treatments had no significant effect on seed number in the field experiment (interaction between cultivar and pollination treatment, F = 1.03, p = 0.395; pollination treatment, F = 0.09, p = 0.907). Seed number did vary between cultivars (cultivar F = 3.461, p = 0.033) with Fury producing a greater number of seeds than Fuego (p = 0.028) but not Vertigo (p = 0.297). There were no significant differences in the total mass of beans per plant between cultivar or pollination treatments (interaction cultivar and treatment, F = 1.19, p = 0.32; pollination, F = 0.24, p = 0.79; cultivar, F = 1.8, p = 0.16; Fig. 1). See Supplementary Material for extended results table.

There were no differences in pod distribution on the stem (e.g. dominant floral node) between pollination treatments or cultivars (interaction pollination treatment and cultivar; F = 1.101, p = 0.357; pollination treatment; F = 0.761, p = 0.469; cultivar; F = 2.732, p = 0.068).

There was again a non-linear relationship between total bean number and total bean mass per plant (model including log(bean number +1) and log(bean mass +1) had the best fit; Fig. 3). The relationship did not vary between the cultivars tested (deletion test for interaction of bean number, pollination treatment and cultivar, F = 1.344, p = 0.25; deletion test for cultivar, F = 0.682, p = 0.507) or pollination treatments (interaction pollination treatment and bean number; F = 1.462, p = 0.215).

**Figure 4:**
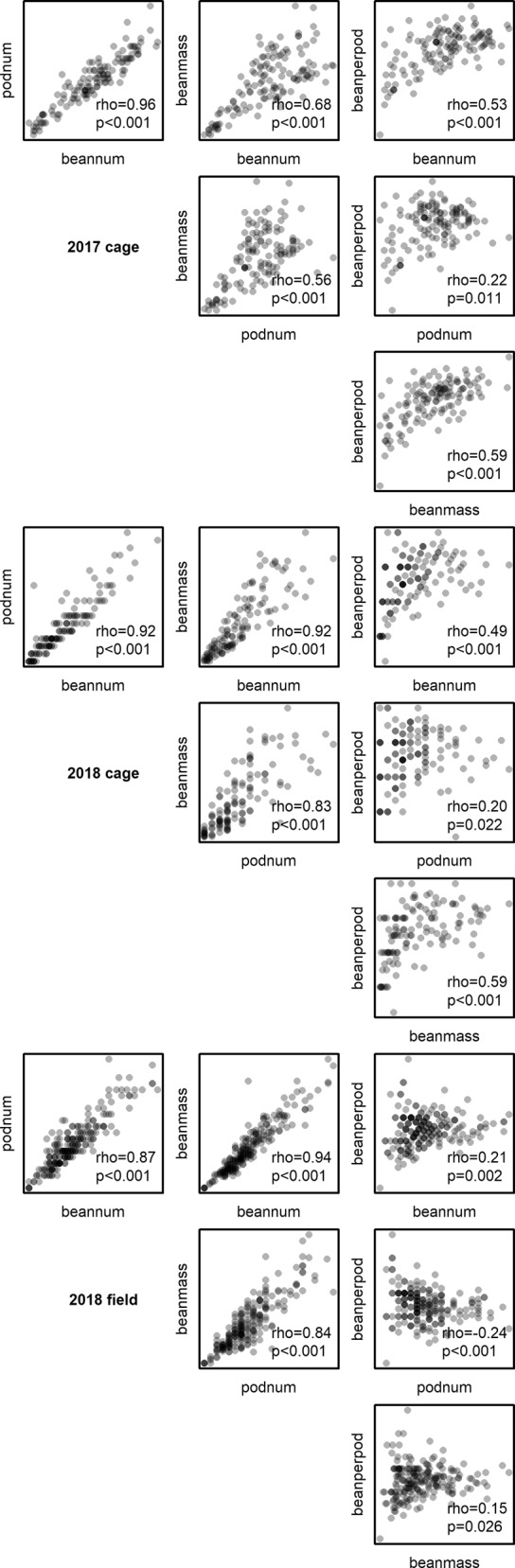
Spearman rank correlation coefficients between variables in current study, split by year and experimental method but across pollination treatments and cultivars.

Correlation coefficients between yield parameters ranged from 0.15 (rho, p = 0.026; bean per pod and bean mass in field experiment) to 0.96 (rho, p < 0.001; pod number and bean number in 2017 cage experiment), correlations between all measured yield parameters are presented in Fig. 4.

### Economic analysis

Pollination dependence varied widely between the cultivars, year, whether the experiment was conducted in cages or in the field, and which yield parameter was used (Table 1). Total bean and pod number per plant showed a wide range of dependence (bean number +75% for Diana07 to −50% for Hedin/2 in 2018; pod number +62% for Diana07 to −55% for Hedin/2 in 2018) while average number of beans per pod, and total bean mass per plant showed a smaller range in dependence between cultivar and treatment combinations. This variation between yield parameters with cultivar and other contextual factors leads to different estimates of the benefits of pollination services for faba bean. Using bean number alone gives an estimate of economic impact of between −£281.27/ha to £425.24/ha, pods alone −£309.14/ha to £351.36/ha, and yield mass −£287.29/ha to £354.69/ha. Extrapolating up to the whole country and assuming only one cultivar is grown, this produced estimates of benefits ranging between −£55.6M (lowest benefit, Hedin/2, pod number) to £76.4M (highest benefit, Diana07, bean number) (Table 2).

**Table 1:** Effect of pollination treatments on yield parameters in faba bean in flight cage and field experiments.

		Bean number				Pod number				Beans per pod			
		Self	Trip	Cross	%	Self	Trip	Cross	%	Self	Trip	Cross	%
2017 cage	Diana07	11 ± 8	37 ± 15	39 ± 19	71*	6 ± 4	15 ± 4	15 ± 7	60*	1.9 ± 0.6	2.5 ± 0.6	2.7 ± 0.4	**27**
	Fuego	23 ± 13	44 ± 13	44 ± 20	47*	9 ± 4	13 ± 4	14 ± 4	**33**	2.6 ± 0.6	3.5 ± 0.2	3.1 ± 1.2	22*
	Fury	26 ± 19	46 ± 13	43 ± 24	**41**	11 ± 6	16 ± 4	14 ± 8	**28**	2.1 ± 1.2	2.9 ± 0.3	3.1 ± 0.7	31*
	Vertigo	33 ± 19	46 ± 14	38 ± 22	**21**	12 ± 5	15 ± 4	12 ± 6	**10**	2.6 ± 0.8	3.1 ± 0.5	3 ± 0.7	**18**
	Hedin/2	68 ± 12	64 ± 16	54 ± 10	−13*	22 ± 5	21 ± 4	19 ± 5	−11*	3 ± 0.4	3 ± 0.4	2.9 ± 0.4	−**2**
2018 cage	Diana07	3 ± 2	9 ± 8	11 ± 4	75*	2 ± 1	5 ± 3	4 ± 2	62*	1.2 ± 0.9	1.9 ± 0.6	2.5 ± 0.4	**45**
	Fuego	9 ± 7	10 ± 5	13 ± 9	22*	4 ± 2	4 ± 2	5 ± 3	**4**	2.5 ± 1.1	2.9 ± 0.9	2.7 ± 1.1	11*
	Fury	9 ± 8	13 ± 13	15 ± 9	**37**	6 ± 4	6 ± 6	6 ± 3	**−4**	1.4 ± 1.1	2.3 ± 0.9	2.7 ± 0.3	46*
	Vertigo	18 ± 14	15 ± 13	21 ± 6	**2**	6 ± 4	5 ± 4	7 ± 2	**6**	2.6 ± 1.2	2.3 ± 1.4	3 ± 0.6	**2**
	Hedin/2	32 ± 13	18 ± 9	14 ± 6	−50*	12 ± 5	6 ± 3	5 ± 1	−55*	2.7 ± 0.4	3.2 ± 0.4	2.7 ± 0.6	**8**
2018 field	Fuego	16 ± 12	16 ± 11	14 ± 7	−**1**	7 ± 5	6 ± 4	6 ± 4	**−5**	2.4 ± 0.9	2.4 ± 1	2.4 ± 1	−**3**
	Fury	21 ± 10	19 ± 9	20 ± 11	−**10**	9 ± 4	9 ± 4	8 ± 5	**0**	2.3 ± 0.6	2.1 ± 0.6	2.6 ± 0.8	−**10**
	Vertigo	16 ± 6	16 ± 11	20 ± 9	**4**	6 ± 3	6 ± 5	8 ± 3	**5**	2.7 ± 0.5	2.5 ± 0.7	2.5 ± 0.5	−**8**

Values presented are rounded mean ± SD and percentage pollination dependence (indicated by %). Pollination dependence is rounded and is calculated from average of hand and tripping treatments in cage experiments, and from tripping treatment in field experiment. In field experiment, cross = open. Asterisks indicate pollination benefit where posthoc test of pollination treatments within a cultivar are significant (p < 0.05).

**Table 2:** Pollination benefit valuation estimates for UK faba bean based upon different cultivars, yield parameters, and experimental methodologies.

Yield mass	Value of pollination per hectare (£/ha)	National UK value of pollination (£millions)
cage 2017	**174.17** (−59.64, 354.69)	**31.3** (−10.7, 63.8)
cage 2018	**76.98** (−287.29, 328.18)	**13.8** (−51.6, 59)
field 2018	−**9.93** (−62.10, 34.97)	−**1.8** (−11.2, 6.3)
**Bean number**		
cage 2017	204.79* (−71.64*, 404.08*)	36.9* (−12.9*, 72.6*)
cage 2018	112.62* (−281.27*, 425.24*)	20.2* (−50.6*, 76.4*)
field 2018	−**57.02** (−13.48, 21.34)	−**10.2** (−2.4, 3.8)
**Pod number**		
cage 2017	**134.03** (−64.39*, 340.40*)	**24.1** (−11.6*, 61.2*)
cage 2018	**10.51** (−309.14*, 351.36*)	**1.9** (−55.6*, 63.2*)
field 2018	**0.95** (−27.06, 27.31)	**0.2** (−4.9, 4.9)
**Beans per pod**		
cage 2017	132.03* (−11.98, 172.84)	23.7* (−2.2, 31.1)
cage 2018	110.86* (10.63, 257.56)	19.9* (1.9, 46.3)
field 2018	−**38.29** (−56.17, −15.48)	−**6.9** (−10.1, −2.8)
Klein *et al*.^31^	**141.4**	**22.8**

Bold numbers are mean across the three commercial cultivars (Fury, Fuego and Vertigo), and numbers in brackets are economic value for the least and most pollination dependent cultivar respectively of the five tested (note in field experiment, only Fury, Fuego and Vertigo were tested). Valuation based on UK 2015–2017 value of £565.61/ha. Estimates based on significant differences between pollination treatments within cultivars (p < 0.05) are indicated with asterisks.

## Discussion

Experimentation over two years in two environments revealed high variability in the pollination dependence of faba bean. In our cage experiment using potted plants, the five cultivars responded differently to pollination treatments. The relative dependence of cultivars generally remained consistent, while in absolute terms yield benefit varied between years and with the yield parameter measured. In our field experiment, the three cultivars tested showed no measurable response to the pollination treatments. Our experimental design ensured that environmental conditions were consistent within each experiment and pollination treatments were consistent across experiments; the results therefore highlight the importance of a number of contextual factors in determining the benefits of pollination.

Differences in the level of pollination dependence between faba bean genotypes have previously been identified in plant breeding studies, where the ability to produce seed without insect pollination is termed autofertility^32,33^. In our study, Hedin/2, an inbred line reputed to be highly autofertile, had no measurable dependence on additional pollination and as discussed below, the pollination treatments actually reduced productive output. There are several heritable traits associated with autofertility relating to the quantity and quality of pollen per flower, angles of the petals and floral organs, and characteristics of the stigma^34,35^. While the highest yields in our study came from commercial cultivars receiving additional pollination treatments (Fig. 1), breeding faba bean to be more autofertile, for instance by using Hedin/2 in breeding programmes, could reduce vulnerability of food producers to unstable pollination services^15,16^. With future advances in breeding (for example to increase maximum yield potential), growers in pollinator-depauperate landscapes may be able to choose such cultivars with particularly low dependence. A simple rank of cultivars based on average yield without additional pollination in our experiments suggests that Vertigo would be a sensible choice for producers if pollination was likely to be limiting. However, between-cultivar comparisons were at times not statistically significant (e.g. for yield mass) and estimates of dependence are likely to differ between contexts, particularly when translating to field conditions.

There was exceptionally high variability in yield and responses to pollination treatments of plants within each treatment combination. Alongside heritable autofertility described above, high levels of autofertility are seen in hybrid faba bean plants, which are typically also capable of greater yield production^25,36^. The number of hybrid plants in a given faba bean population is typically around 35%^37^ but this can change depending on the levels of cross fertilization in previous generations^34^. Random differences in the proportion of hybrid individuals in each sample of experimental plants may partially explain the high variability within and between some of our treatment combinations. However, if heterosis was the key factor contributing variability we would expect the two inbred breeding lines, Diana07 and Hedin/2 to contain few if any hybrid individuals and therefore show the least variability, yet this is not the case (see Supplementary Information).

There were no measurable effects of pollination treatments in our field experiment. Yield benefits of insect pollination to faba bean in the field are known to vary with season^27^ and site^38,39^, suggesting important roles of pollinator populations, soil and weather conditions. We conducted the same manual pollination treatments in the cage and field environments so it is highly unlikely that differences in pollination effort (as may be expected if there were differences in pollinator density or species) caused differences within and between our field and flight cage experiments. Similarly, weather conditions are unlikely to have made the plants unresponsive to pollination treatments in the field experiment. In contrast to the cage experiments, plants in the field were not irrigated. This water limitation would be expected to reduce autofertility and make plants more dependent on outside intervention^16,29^. Now, if the yield of plants grown in the field was restricted by some other factor (e.g. pests, pathogens, or nutrients^11^) that we did not quantify, the effects of pollination treatments may have been masked. For example, if plants only had the resources available to take 50% of pods through to maturity, we would be unable to distinguish a plant that was 50% autofertile from a plant that was 70 or 100% autofertile, as the additional pods would be aborted or fail to develop^36^. There were indications of resource limitation in all experiments and in 2018, more pods and beans were produced in the field than the cage experiment but this did not translate into a greater average yield mass.

We simulated insect pollination via hand pollination so that all plants in an experiment could be grown in the same enclosure and be subjected to identical agronomic and environmental conditions. If not controlled for, enclosures used to exclude insect pollinators can interfere with plant growth^25,39^ and therefore may under- or over-estimate the effects of pollination. However, the cultivar Hedin/2 actually lost yield as a result of pollination treatments in this study, suggesting that the pollination treatments necessary for our experimental design stymied yield production in at least some of the plants. In addition to effects of plant genotype, the benefits of pollination are known to vary with species of pollinator^24,40^ perhaps due to the precise timing of pollination, or amount of pollen transferred, but we found no differences in efficacy of hand vs insect pollination in this (e.g. open pollinated vs tripping in field experiment) or previous research^39^. Losses in faba bean yield as a result of insect pollination have been documented previously but not discussed in detail^30^. Yield losses due to excess pollination have been reported in horticultural crops, with tissue damage thought to lead to fruit malformation (over-pollination^41^). Methods proposed by Link^36^ offer a clearer way of assessing autofertility which avoids disturbing the experimental flowers (manually reducing flower number with assumption that any fertilized flowers will develop into pods), but we are then left with difficulty understanding how this translates to yield production in the field or what the benefits of insect pollination are likely to be. Field level assessment of pollination could be carried out, even by growers themselves^42^ to help establish their pollination need based on cultivar and context. This is labour intensive, particularly if whole plant manipulations are required (see below), and so growers would likely require support.

Our results suggest that within-plant measurements of yield are unlikely to be representative of total yield across the whole stem. Diana07 showed a significant difference between the dominant floral node under selfing and pollination treatments. Studies measuring yield on a fixed subset of floral nodes within a stem therefore may not accurately represent pollination dependence. The commercial cultivars we tested shared a distribution of yield that is characteristic of faba bean with the main grouping of pods approximately one third of the way up the main stem^29,33^. Hedin/2 appeared to have a different distribution of yield (Fig. 2) indicating resource limitation, with greatest podset on the lowest floral nodes and an apparent reduction in podset higher up the plants due to competition with previously set pods^43^. Practical ways to account for these between-cultivar differences are either to measure yield across the whole stem, or to take samples from more than one location on the stem.

We have shown that the relationship between some yield parameters is non-linear and can vary with pollination treatment and cultivar, while correlations between different yield parameters vary from weak to strong. To get the most accurate assessment of pollination dependence of a cultivar within a certain context then a whole plant manipulation is required with subsequent measurement of final marketable yield. Labour costs for this may however be prohibitive, particularly if multiple fields and cultivars are to be assessed, in which case, the selection of alternative pollination response metrics needs to be data-driven and validated across multiple contexts. We have shown that in faba bean, seed number is consistently better correlated with final yield than pod number or bean number per pod. While we present yield data across several parameters, this information is not consistently presented in studies of pollination benefits (e.g. in faba bean, Bartomeus *et al*.^23^ present only yield mass and protein content; Suso & del Río^30^ present bean and pod numbers but not mass). Desk-based studies that collate information of yield benefits from across a range of literature sources are likely to find very different estimates of pollination benefit and economic value depending upon the cultivars, yield parameters measured, and assessment methodologies used in the available papers and need to account for this.

It is possible to make a wide range of valuations using our experimental results. Translating the observed differences in pollination service found in this study alone into economic terms produces UK-wide value estimates that range from −£55.6M to £76.4M depending on the cultivar, year, assessment method, and yield parameter used to estimate benefits (Table 2). This low consistency of pollination dependence even within the same experimental framework brings into question the robustness of current economic valuations of insect pollination services^44^ and the calculation of cost-benefit and cost-efficiency ratios for different farm insect pollination management methods.

In summary, our results highlight the need for changes in pollinator dependence testing and bring new evidence to the narrative that benefits of insect pollination are highly variable and context dependent^12^. We must assume that single aggregated per-crop measures of pollinator dependence are not sufficient to allow individual producers to match cultivar selection or investments in pollinator-friendly management with positive yield outcomes; on-farm assessments^42^ are more likely to be useful in this regard, though producer context itself is not fixed due to increasing between-year climate variability. Because of the variable and at times non-linear within-plant relationships we have demonstrated, it is important that experimental studies quantifying crop pollination dependence in economic terms report yield parameters of direct economic relevance (marketable crop output) at a whole plant or larger scale. The large variation between yield parameters and experimental methods we have shown, demands that literature-based estimates of the economic benefits of pollinators focus upon marketable crop outputs (or parameters known to correlate strongly) and draw upon as much available data as possible including (and accounting for) multiple cultivars, sites, years and assessment methods. Regarding pollination dependence, cultivar choice is clearly important, but it is moderated by many factors that are difficult to summarise in a single experiment. Given the important yield impacts that pollination can have in some cultivars and situations, we suggest that pollination dependence is routinely assessed via industry-funded multi-year, multi-site agronomic trials within the framework of crop levy board recommended-lists.

## Methods

### Cage experiments

Flight cage experiments were conducted in summer 2017 and 2018 using a completely randomised design to test whether five spring cultivars of faba bean (*Vicia faba* L.) differ in their requirement for insect pollination. Insect pollination was simulated by hand to ensure that pollination effort was equal across all plants and that conditions were otherwise identical with all plants housed in a single mesh cage (dimensions 5 × 5 × 2 m constructed from 1.33 mm aperture mesh). Plants were grown to maturity in 4 l pots of 20 cm diameter in John Innes no. 2 compost and were supported by bamboo canes. Pots were raised 100 mm above ground upon a metal grid to allow free-draining and were positioned in 5 rows spaced 500 mm apart. There was a target of 10 replicate plants per cultivar and treatment combination, but this was limited to 6 plants for some combinations due to inconsistent germination (Supplementary Information). Plants were irrigated by drip irrigation which was applied variably depending upon weather conditions. The cage was located at the Crop and Environment Laboratory, University of Reading (51 44′N, 00 94′E).

Three pollination treatments (hand pollination, tripping and selfing) and their effects on yield parameters for each cultivar was measured. The three treatments simulate the three pollination mechanisms of faba bean a) cross-pollination by bees, b) tripping (self-pollination facilitated by bees) or c) autofertility (spontaneous self-pollination without bees^45^). For cross-pollination (hand pollination), the lowest 3 flowers on each floral node of the primary stem were hand pollinated on at least 2 occasions per flower by manually pulling apart the flag and wing petals and adding pollen with a paint brush. For tripping, equivalent flowers were tripped on at least 2 occasions per flower by manually pulling apart the flag and wing petals. Hand pollination and tripping treatments were continued until each experimental plant stopped producing flowers on the primary stem. For self-pollination, plants were left undisturbed for the duration of the experiment. Pollen for hand pollination was collected fresh, immediately prior to the hand pollination treatment from a nearby patch of faba beans of cultivar Babylon. Hand simulation of insect pollination is an effective method: previous work found no differences between insect pollination, insect pollination plus tripping, or exclusion from pollinators plus tripping^39^. Treatments were applied to the lowest 3 flowers of each floral node only following two years of experimentation using cultivar Wizard in which 88–94% of pods were set on these first 3 raceme positions (unpublished data).

The cultivars tested comprised three commercial cultivars, an inbred line reputed to be highly autofertile (Hedin/2), and a highly inbred line reputed to have low autofertility (Diana07). Commercial cultivars came directly from the breeders and were selected to broadly represent the range of UK commercial cultivars and the range of breeding strategies employed. Vertigo, Fuego and Fury all had prominent market share in the UK as of 2017. Fuego is a synthetic cultivar (a population derived from crosses between several inbred lines) sold by Limagrain UK (www.lgseeds.co.uk). Vertigo is a synthetic cultivar, while Fury is an inbred line, both are sold by LS Plant Breeding (www.lspb.eu). For Vertigo and Fury, new seed stock from the breeders was used each year, for other cultivars the same seed stock was used in both years.

At maturity, we harvested each pod from each plant individually and recorded on which floral node position the pod occurred. The bean pods were dried until constant mass (oven dried at 80 °C in 2017, air dried at room temperature in 2018 to retain seed viability for future work) before recording the mass and number of beans in each pod. Where plants had more than one stem we focused on the primary stem upon which treatments took place.

### Field experiment

A field experiment was conducted in summer 2018 using a randomised block design to compare pollination requirements of the three commercial faba bean cultivars, Fuego, Fury and Vertigo. Seeds were sown in April 2018 at a rate of 40 seeds per m^2^, in plots measuring 1.8 m by 6 m. There were 8 replicate blocks separated by a wheat buffer. The experiment took place at the University of Reading experimental farm (51 48′N, 00 89′E).

A 1 m^2^ area in the middle of each plot was excluded from insect pollinators by a mesh cage that was in place during the flowering period. Mesh netting of 2 mm aperture was supported by metal rebar and 20 mm plastic tubing in each corner, resulting in a straight-sided cage approximately 1.5 m tall that we sealed to the ground on each side with tent pegs.

Field experiment pollination treatments were either selfing, tripping, or an open pollination treatment. Based upon results of the 2017 cage experiment and others, which showed little difference between tripping and cross pollination treatments^39^, we tested only the effect of tripping. Within each cage, three plants were allocated to the tripping treatment. As in the cage experiments, the lowest 3 flowers on each floral node of the primary stem were tripped on at least 2 occasions per flower by manually pulling apart the flag and wing petals. Three other plants in each cage were allocated to the selfing treatment and were left undisturbed for the duration of the field experiment. Three plants per plot outside of the cages were randomly allocated to the open pollination treatment, these plants were not excluded from pollinators and therefore represent open field pollination (without effect of enclosure controlled for).

At maturity, we recorded how many pods occurred at each floral node position. Plants were then taken and bagged whole from the field. Plants and their pods were oven dried until constant mass as 80 °C before recording bean mass, bean number and number of pods per plant. Again, where plants had more than one stem we harvested only the primary stem upon which treatments took place.

### Statistical analysis

The experiments were primarily analysed using ANOVA in R statistical software (version 3.5.2). ANOVA models included an interaction term between cultivar and pollination treatment to test whether effect of pollination treatment varied between cultivars. For the cage experiment, to test whether patterns changed between years, we included a three-way interaction between cultivar, pollination treatment and year, and all lower level interactions. For the field experiment, we included blocks as a random effect. Post-hoc Tukey tests were used where significant effects were detected; before the post-hoc tests were applied the model was simplified to only include significant terms (p < 0.05). The model residuals were checked that they met model assumptions. Bean mass and pod number required log transformation.

Relationships between the yield parameters of bean number and total bean mass per plant were analysed using linear regression models. The significance of parameters was tested with deletion tests. The linear regression model residuals were checked to ensure they met model assumptions. Bean number and total bean mass were both log transformed in the final models. In addition, correlation coefficients between all yield parameters (data split by experiment but grouped across cultivars and pollination treatments) were calculated using Spearman’s rank correlation.

To calculate how yield was allocated across the floral nodes of plants in different treatments, the dominant floral node was identified using local polynomial regressions (loess) which were run to produce a smoothed curve of pod number against floral nodes of each plant. The floral node with the maximum (model predicted) pod number was recorded for each plant and this was used as a yield parameter in ANOVA as above.

### Economic analysis

Our economic analysis is based upon the observed pollination dependence, measured as ((mean yield of plants receiving pollination – mean yield of plants without pollination)/mean yield of plants receiving pollination), for different yield parameters, cultivars, and experiments. Where the pollination treatment caused a yield reduction, we reversed this equation to quantify proportion of yield lost due to pollination. For plants receiving pollination we used the average of plants receiving tripping and hand-pollination treatments (cage experiments) or tripping treatments only (field experiment), and for plants without pollination we used plants receiving the selfing treatment. We use the average of 2015–2017 UK-wide values from the 2018 Agriculture in the UK report^46^ to control for short term supply and demand fluctuations. The average total market value of the UK’s faba bean crop was £101,665,319, grown over an average of 179,746 ha, equating to £565.61/ha.

## Supplementary Information

Supplementary Information.

## Data Availability

Data supporting the results reported in this paper are openly available at 10.6084/m9.figshare.11742975.v1.
