# Supplementary material for: Yield benefits of additional pollination to faba bean vary with cultivar, scale, yield parameter and experimental method
Source: Sci Rep. 2020 Feb 7;10:2102. doi: 10.1038/s41598-020-58518-1 (PMC7005869; doi:10.1038/s41598-020-58518-1)
Supplement: Supplementary file 1 — Supplementary Information. [file 41598_2020_58518_MOESM1_ESM.pdf]

Supplementary Information - Yield benefits of additional pollination to faba bean vary with cultivar, scale, yield parameter and experimental method - Bishop, J., Garratt, M.P.D. & Breeze, T.D.

Table S1: Number of replicate plants, cage experiment

	Treatment	2017	2018
Diana	hand	7	10
	self	10	10
	trip	9	10
Fuego	hand	9	10
	self	10	9
	trip	7	10
Fury	hand	7	6
	self	6	6
	trip	8	6
Hedin	hand	10	10
	self	10	11
	trip	10	10
Vertigo	hand	10	10
	self	10	10
	trip	10	10
Grand Total		159	153

Table S2: Coefficient of variation within treatment combinations

		**Bean number**			**Pod number**			**Beans per pod**		
		Auto	Trip	Cross	Auto	Trip	Cross	Auto	Trip	Cross
**2017 cage**	Diana07	0.738	0.4	0.479	0.708	0.3	0.492	0.327	0.237	0.151
	Fuego	0.561	0.297	0.457	0.481	0.294	0.275	0.214	0.062	0.383
	Fury	0.715	0.282	0.563	0.519	0.229	0.539	0.592	0.113	0.24
	Vertigo	0.564	0.299	0.582	0.423	0.246	0.526	0.332	0.162	0.221
	Hedin/2	0.181	0.248	0.186	0.204	0.178	0.254	0.122	0.132	0.12
**2018 cage**	Diana07	0.768	0.833	0.423	0.735	0.735	0.396	0.764	0.331	0.162
	Fuego	0.737	0.529	0.681	0.537	0.438	0.673	0.437	0.319	0.411
	Fury	0.866	1.022	0.614	0.747	1.065	0.598	0.819	0.38	0.121
	Vertigo	0.8	0.852	0.279	0.754	0.784	0.243	0.45	0.604	0.204
	Hedin/2	0.412	0.459	0.419	0.412	0.476	0.28	0.144	0.131	0.216
**2018 field**	Fuego	0.722	0.701	0.524	0.682	0.625	0.607	0.389	0.417	0.391
	Fury	0.508	0.49	0.555	0.494	0.397	0.554	0.266	0.264	0.319
	Vertigo	0.383	0.685	0.476	0.428	0.716	0.438	0.182	0.3	0.192

Table S3: Tukey multiple comparisons of means for cage experiment, bean mass (95% family-wise confidence level)

Fit: aov(formula = log(beanmass + 1) ~ cv + poll2 + year + cv:poll2 + year:poll2, data = data3)

Statistical significance of model parameters
Poll:cv:year (F=0.809, p=0.521)
Poll:cv (F=11.366, p<0.001)
Poll:year (F=17.809, p<0.001)
Cv:year (F=6.242, p<0.001)

$cv	diff	lwr	upr	p
Fuego-Diana	0.798071	0.465606	1.130535	0
Fury-Diana	0.741793	0.378573	1.105013	5E-07
Vertigo-Diana	1.092137	0.76265	1.421623	0
Hedin-Diana	0.874977	0.552163	1.197791	0
Fury-Fuego	-0.05628	-0.41656	0.304004	0.992888
Vertigo-Fuego	0.294066	-0.03218	0.620311	0.099222
Hedin-Fuego	0.076907	-0.2426	0.396411	0.964333
Vertigo-Fury	0.350344	-0.00719	0.70788	0.057885
Hedin-Fury	0.133185	-0.21821	0.484581	0.835732
Hedin-Vertigo	-0.21716	-0.53356	0.099246	0.32751

$poll2	diff	lwr	upr	p
pollinated-self	0.183571	0.02298	0.344162	0.025238

$year	diff	lwr	upr	p
2018cage-2017cage	-0.78989	-0.9415	-0.63828	0

$`cv:poll2`	diff	lwr	upr	p
Fuego:self-Diana:self	0.996702	0.339239	1.654166	9.87E-05
Fury:self-Diana:self	0.814987	0.052943	1.577031	0.025443
Vertigo:self-Diana:self	1.348537	0.682505	2.014569	0
Hedin:self-Diana:self	1.514624	0.872115	2.157133	0
Diana:pollinated-Diana:self	0.577869	-0.00711	1.162852	0.055933
Fuego:pollinated-Diana:self	1.287602	0.702619	1.872584	0
Fury:pollinated-Diana:self	1.254171	0.644435	1.863907	0
Vertigo:pollinated-Diana:self	1.540064	0.963041	2.117088	0
Hedin:pollinated-Diana:self	1.123238	0.550892	1.695585	1E-07
Fury:self-Fuego:self	-0.18171	-0.92784	0.564406	0.99883
Vertigo:self-Fuego:self	0.351835	-0.29592	0.999588	0.77477
Hedin:self-Fuego:self	0.517922	-0.10562	1.141463	0.199584
Diana:pollinated-Fuego:self	-0.41883	-0.98292	0.14525	0.348029
Fuego:pollinated-Fuego:self	0.290899	-0.27318	0.854983	0.823613
Fury:pollinated-Fuego:self	0.257469	-0.33225	0.847183	0.928139
Vertigo:pollinated-Fuego:self	0.543362	-0.01246	1.099187	0.061395
Hedin:pollinated-Fuego:self	0.126536	-0.42443	0.677504	0.999268
Vertigo:self-Fury:self	0.53355	-0.22013	1.287232	0.418731
Hedin:self-Fury:self	0.699637	-0.03334	1.432615	0.075322
Diana:pollinated-Fury:self	-0.23712	-0.92023	0.445994	0.983514
Fuego:pollinated-Fury:self	0.472614	-0.2105	1.155727	0.45333
Fury:pollinated-Fury:self	0.439184	-0.26524	1.14361	0.606323
Vertigo:pollinated-Fury:self	0.725077	0.048768	1.401386	0.024771
Hedin:pollinated-Fury:self	0.308251	-0.36407	0.980574	0.904863
Hedin:self-Vertigo:self	0.166087	-0.46648	0.798656	0.997891
Diana:pollinated-Vertigo:self	-0.77067	-1.34472	-0.19662	0.001068
Fuego:pollinated-Vertigo:self	-0.06094	-0.63498	0.513112	0.999999
Fury:pollinated-Vertigo:self	-0.09437	-0.69362	0.504886	0.999968
Vertigo:pollinated-Vertigo:self	0.191527	-0.37441	0.757461	0.98618
Hedin:pollinated-Vertigo:self	-0.2253	-0.78646	0.335866	0.956853
Diana:pollinated-Hedin:self	-0.93676	-1.48334	-0.39017	4.8E-06
Fuego:pollinated-Hedin:self	-0.22702	-0.7736	0.319558	0.946726
Fury:pollinated-Hedin:self	-0.26045	-0.83345	0.312542	0.90937
Vertigo:pollinated-Hedin:self	0.02544	-0.51261	0.563493	1
Hedin:pollinated-Hedin:self	-0.39139	-0.92442	0.141649	0.364348
Fuego:pollinated-Diana:pollinated	0.709732	0.232096	1.187369	0.000152
Fury:pollinated-Diana:pollinated	0.676302	0.168651	1.183953	0.00122
Vertigo:pollinated-Diana:pollinated	0.962195	0.494341	1.430049	0
Hedin:pollinated-Diana:pollinated	0.545369	0.083296	1.007443	0.007719
Fury:pollinated-Fuego:pollinated	-0.03343	-0.54108	0.47422	1
Vertigo:pollinated-Fuego:pollinated	0.252463	-0.21539	0.720317	0.781393
Hedin:pollinated-Fuego:pollinated	-0.16436	-0.62644	0.297711	0.980502
Vertigo:pollinated-Fury:pollinated	0.285893	-0.21256	0.784352	0.714594
Hedin:pollinated-Fury:pollinated	-0.13093	-0.62397	0.362104	0.997697
Hedin:pollinated-Vertigo:pollinated	-0.41683	-0.86878	0.035129	0.099068

$`poll2:year`	diff	lwr	upr	p

pollinated:2017cage-self:2017cage	0.393292	0.101874	0.68471	0.003182
self:2018cage-self:2017cage	-0.53179	-0.87669	-0.18689	0.000507
pollinated:2018cage-self:2017cage	-0.52622	-0.81823	-0.2342	3.05E-05
self:2018cage-pollinated:2017cage	-0.92508	-1.23041	-0.61976	0
pollinated:2018cage-pollinated:2017cage	-0.91951	-1.16351	-0.6755	0
pollinated:2018cage-self:2018cage	0.005574	-0.30032	0.311469	0.999962

Table S4: Tukey multiple comparisons of means for cage experiment, bean number (95% family-wise confidence level)

Fit: aov(formula = beannum ~ cv + poll2 + year + cv:poll2 + cv:year + year:poll2, data = data3)

Statistical significance of tested model parameters
Poll:cv:year (F=0.809, p=0.521)
Poll:cv (F=11.366, p<0.001)
Poll:year (F=17.809, p<0.001)
Cv:year (F=6.242, p<0.001)

$cv	diff	lwr	upr	p
Fuego-Diana	5.943396	-0.76117	12.64796	0.10925
Fury-Diana	9.752731	2.416306	17.08916	0.0029
Vertigo-Diana	12.08606	5.500174	18.67195	8.8E-06
Hedin-Diana	23.60346	17.12245	30.08448	0
Fury-Fuego	3.809335	-3.52709	11.14576	0.610891
Vertigo-Fuego	6.142668	-0.44322	12.72856	0.08053
Hedin-Fuego	17.66007	11.17905	24.14108	0
Vertigo-Fury	2.333333	-4.8948	9.561466	0.901473
Hedin-Fury	13.85073	6.718026	20.98344	2.1E-06
Hedin-Vertigo	11.5174	5.15923	17.87557	1.19E-05

$poll2	diff	lwr	upr	p
pollinated-self	5.14216	1.897119	8.3872	0.002017

$year	diff	lwr	upr	p
2018cage-2017cage	-26.7896	-29.8462	-23.733	0

$`cv:poll2`	diff	lwr	upr	p
Fuego:self-Diana:self	9.867442	-3.5204	23.25528	0.35883
Fury:self-Diana:self	14.06278	-1.45461	29.58017	0.112931
Vertigo:self-Diana:self	21.46898	8.081139	34.85682	2.69E-05
Hedin:self-Diana:self	43.95012	30.86679	57.03344	0
Diana:pollinated-Diana:self	17.36777	5.566663	29.16888	0.000186
Fuego:pollinated-Diana:self	22.13995	10.22803	34.05187	4E-07
Fury:pollinated-Diana:self	24.59994	12.18397	37.0159	1E-07
Vertigo:pollinated-Diana:self	25.09007	13.389	36.79114	0
Hedin:pollinated-Diana:self	30.91186	19.30156	42.52216	0
Fury:self-Fuego:self	4.195335	-10.9978	19.38849	0.996894
Vertigo:self-Fuego:self	11.60154	-1.40911	24.61218	0.126817
Hedin:self-Fuego:self	34.08267	21.38559	46.77976	0
Diana:pollinated-Fuego:self	7.500327	-3.87107	18.87172	0.525037
Fuego:pollinated-Fuego:self	12.27251	0.786154	23.75886	0.025709
Fury:pollinated-Fuego:self	14.73249	2.724216	26.74077	0.004491
Vertigo:pollinated-Fuego:self	15.22263	3.955082	26.49017	0.000956
Hedin:pollinated-Fuego:self	21.04442	9.871162	32.21767	3E-07
Vertigo:self-Fury:self	7.406202	-7.78695	22.59936	0.867393
Hedin:self-Fury:self	29.88734	14.96182	44.81286	0
Diana:pollinated-Fury:self	3.304992	-10.5104	17.12034	0.998986
Fuego:pollinated-Fury:self	8.077173	-5.83295	21.9873	0.699798
Fury:pollinated-Fury:self	10.53716	-3.80697	24.88129	0.36371
Vertigo:pollinated-Fury:self	11.02729	-2.7027	24.75729	0.240422
Hedin:pollinated-Fury:self	16.84908	3.19636	30.50181	0.00412
Hedin:self-Vertigo:self	22.48114	9.784051	35.17822	1.9E-06
Diana:pollinated-Vertigo:self	-4.10121	-15.4726	7.270187	0.978591
Fuego:pollinated-Vertigo:self	0.670971	-10.8154	12.15733	1
Fury:pollinated-Vertigo:self	3.130958	-8.87732	15.13924	0.998004
Vertigo:pollinated-Vertigo:self	3.621091	-7.64646	14.88864	0.990428
Hedin:pollinated-Vertigo:self	9.442881	-1.73037	20.61614	0.180083
Diana:pollinated-Hedin:self	-26.5823	-37.5936	-15.5711	0
Fuego:pollinated-Hedin:self	-21.8102	-32.9401	-10.6802	1E-07
Fury:pollinated-Hedin:self	-19.3502	-31.018	-7.68236	1.16E-05
Vertigo:pollinated-Hedin:self	-18.86	-29.764	-7.95607	3.7E-06
Hedin:pollinated-Hedin:self	-13.0383	-23.8448	-2.23174	0.005704
Fuego:pollinated-Diana:pollinated	4.77218	-4.81782	14.36219	0.852467
Fury:pollinated-Diana:pollinated	7.232167	-2.97717	17.44151	0.417791
Vertigo:pollinated-Diana:pollinated	7.722301	-1.60451	17.04912	0.203314
Hedin:pollinated-Diana:pollinated	13.54409	4.331409	22.75677	0.00019
Fury:pollinated-Fuego:pollinated	2.459987	-7.87724	12.79722	0.999027
Vertigo:pollinated-Fuego:pollinated	2.95012	-6.51651	12.41675	0.992339
Hedin:pollinated-Fuego:pollinated	8.77191	-0.5823	18.12612	0.087011
Vertigo:pollinated-Fury:pollinated	0.490133	-9.60341	10.58367	1
Hedin:pollinated-Fury:pollinated	6.311923	-3.67625	16.3001	0.58734
Hedin:pollinated-Vertigo:pollinated	5.82179	-3.2624	14.90598	0.567212

$`cv:year`	diff	lwr	upr	p

Fuego:2017cage-Diana:2017cage	9.412391	-1.82043	20.64522	0.189581
Fury:2017cage-Diana:2017cage	10.64389	-1.12169	22.40946	0.114391
Vertigo:2017cage-Diana:2017cage	10.38908	-0.35594	21.1341	0.067566
Hedin:2017cage-Diana:2017cage	33.21406	22.46904	43.95909	0
Diana:2018cage-Diana:2017cage	-20.9943	-32.013	-9.97562	2E-07
Fuego:2018cage-Diana:2017cage	-17.3984	-28.3202	-6.47669	3.16E-05
Fury:2018cage-Diana:2017cage	-15.2551	-27.7629	-2.74718	0.004892
Vertigo:2018cage-Diana:2017cage	-8.60718	-19.6259	2.411511	0.276792
Hedin:2018cage-Diana:2017cage	-6.74254	-17.4068	3.921718	0.586628
Fury:2017cage-Fuego:2017cage	1.231496	-10.6388	13.10174	0.999999
Vertigo:2017cage-Fuego:2017cage	0.976687	-9.88285	11.83622	1
Hedin:2017cage-Fuego:2017cage	23.80167	12.94214	34.66121	0
Diana:2018cage-Fuego:2017cage	-30.4067	-41.5371	-19.2763	0
Fuego:2018cage-Fuego:2017cage	-26.8108	-37.8452	-15.7764	0
Fury:2018cage-Fuego:2017cage	-24.6674	-37.2738	-12.0611	1E-07
Vertigo:2018cage-Fuego:2017cage	-18.0196	-29.15	-6.88918	2.13E-05
Hedin:2018cage-Fuego:2017cage	-16.1549	-26.9346	-5.3753	0.000127
Vertigo:2017cage-Fury:2017cage	-0.25481	-11.6645	11.15492	1
Hedin:2017cage-Fury:2017cage	22.57018	11.16045	33.97991	1E-07
Diana:2018cage-Fury:2017cage	-31.6382	-43.306	-19.9704	0
Fuego:2018cage-Fury:2017cage	-28.0423	-39.6186	-16.466	0
Fury:2018cage-Fury:2017cage	-25.8989	-38.9823	-12.8156	1E-07
Vertigo:2018cage-Fury:2017cage	-19.2511	-30.9189	-7.58325	1.32E-05
Hedin:2018cage-Fury:2017cage	-17.3864	-28.7201	-6.05272	7.58E-05
Hedin:2017cage-Vertigo:2017cage	22.82499	12.47083	33.17915	0
Diana:2018cage-Vertigo:2017cage	-31.3834	-42.0213	-20.7455	0
Fuego:2018cage-Vertigo:2017cage	-27.7875	-38.3249	-17.2501	0
Fury:2018cage-Vertigo:2017cage	-25.6441	-37.8179	-13.4704	0
Vertigo:2018cage-Vertigo:2017cage	-18.9963	-29.6341	-8.35837	1.5E-06
Hedin:2018cage-Vertigo:2017cage	-17.1316	-27.4019	-6.86129	0.00001
Diana:2018cage-Hedin:2017cage	-54.2084	-64.8463	-43.5705	0
Fuego:2018cage-Hedin:2017cage	-50.6125	-61.1499	-40.0751	0
Fury:2018cage-Hedin:2017cage	-48.4691	-60.6429	-36.2954	0
Vertigo:2018cage-Hedin:2017cage	-41.8212	-52.4591	-31.1834	0
Hedin:2018cage-Hedin:2017cage	-39.9566	-50.2269	-29.6863	0
Fuego:2018cage-Diana:2018cage	3.595876	-7.22048	14.41223	0.987814
Fury:2018cage-Diana:2018cage	5.739251	-6.67672	18.15522	0.900455
Vertigo:2018cage-Diana:2018cage	12.38713	1.472885	23.30137	0.012778
Hedin:2018cage-Diana:2018cage	14.25177	3.69547	24.80807	0.000968
Fury:2018cage-Fuego:2018cage	2.143375	-10.1866	14.47338	0.999927
Vertigo:2018cage-Fuego:2018cage	8.791253	-2.0251	19.60761	0.225541
Hedin:2018cage-Fuego:2018cage	10.6559	0.200832	21.11096	0.041725
Vertigo:2018cage-Fury:2018cage	6.647878	-5.76809	19.06384	0.789146
Hedin:2018cage-Fury:2018cage	8.512521	-3.59	20.61504	0.42849
Hedin:2018cage-Vertigo:2018cage	1.864643	-8.69166	12.42094	0.999916

$`poll2:year`	diff	lwr	upr	p
pollinated:2017cage-self:2017cage	12.79471	6.860547	18.72887	4E-07
self:2018cage-self:2017cage	-17.616	-24.5933	-10.6387	0
pollinated:2018cage-self:2017cage	-18.5762	-24.4754	-12.677	0
self:2018cage-pollinated:2017cage	-30.4107	-36.5761	-24.2453	0
pollinated:2018cage-pollinated:2017cage	-31.3709	-36.2831	-26.4587	0
pollinated:2018cage-self:2018cage	-0.96019	-7.09195	5.171574	0.977494

Table S5: Tukey multiple comparisons of means for cage experiment, beans per pod (95% family-wise confidence level)

Fit: aov(formula = beanperpod ~ cv + poll2 + year + cv:poll2, data = data3)

Statistical significance of tested model parameters
poll:cv:year (F= 1.066, p=0.374)
poll:cv (F=2.686, p=0.032)
poll:year (F= 1.154, p=0.284)
cv:year (F=0.981, p=0.419)
year (F=9.822, p=0.002)

$cv	diff	lwr	upr	p
Fuego-Diana	0.756158	0.424977	1.087339	0
Fury-Diana	0.290294	-0.0721	0.652687	0.182677
Vertigo-Diana	0.711055	0.385736	1.036374	1E-07
Hedin-Diana	0.711266	0.391128	1.031405	0
Fury-Fuego	-0.46586	-0.82826	-0.10347	0.004431
Vertigo-Fuego	-0.0451	-0.37042	0.280215	0.995508
Hedin-Fuego	-0.04489	-0.36503	0.275247	0.995307
Vertigo-Fury	0.420761	0.063717	0.777804	0.011823
Hedin-Fury	0.420972	0.068643	0.773302	0.010232
Hedin-Vertigo	0.000212	-0.31386	0.314282	1

$poll2	diff	lwr	upr	p
pollinated-self	0.433507	0.273206	0.593808	2.00E-07

$year	diff	lwr	upr	p
2018cage-2017cage	-0.23959	-0.39058	-0.0886	0.001987

$`cv:poll2`	diff	lwr	upr	p
Fuego:self-Diana:self	0.75737	0.096084	1.418657	0.0114
Fury:self-Diana:self	0.042349	-0.72413	0.808824	1
Vertigo:self-Diana:self	0.913831	0.252544	1.575118	0.000634
Hedin:self-Diana:self	1.037662	0.391416	1.683908	2.58E-05
Diana:pollinated-Diana:self	0.561714	-0.0212	1.144625	0.069541
Fuego:pollinated-Diana:self	1.350236	0.761852	1.938621	0
Fury:pollinated-Diana:self	0.928293	0.315011	1.541574	0.000101
Vertigo:pollinated-Diana:self	1.181978	0.604009	1.759948	0
Hedin:pollinated-Diana:self	1.121759	0.548273	1.695245	1E-07
Fury:self-Fuego:self	-0.71502	-1.46548	0.035438	0.076486
Vertigo:self-Fuego:self	0.156461	-0.48619	0.799116	0.998837
Hedin:self-Fuego:self	0.280292	-0.34688	0.907459	0.917838
Diana:pollinated-Fuego:self	-0.19566	-0.75734	0.366029	0.983147
Fuego:pollinated-Fuego:self	0.592866	0.025502	1.16023	0.032503
Fury:pollinated-Fuego:self	0.170923	-0.42222	0.764066	0.995718
Vertigo:pollinated-Fuego:self	0.424608	-0.13195	0.981164	0.309434
Hedin:pollinated-Fuego:self	0.364389	-0.18751	0.916287	0.523768
Vertigo:self-Fury:self	0.871482	0.121022	1.621942	0.009549
Hedin:self-Fury:self	0.995313	0.258073	1.732553	0.000965
Diana:pollinated-Fury:self	0.519365	-0.16304	1.201768	0.31283
Fuego:pollinated-Fury:self	1.307887	0.620802	1.994972	2E-07
Fury:pollinated-Fury:self	0.885944	0.177422	1.594466	0.003381
Vertigo:pollinated-Fury:self	1.139629	0.461442	1.817817	8.1E-06
Hedin:pollinated-Fury:self	1.07941	0.40504	1.753781	2.79E-05
Hedin:self-Vertigo:self	0.123831	-0.50334	0.750998	0.999788
Diana:pollinated-Vertigo:self	-0.35212	-0.9138	0.209568	0.598922
Fuego:pollinated-Vertigo:self	0.436405	-0.13096	1.003769	0.298035
Fury:pollinated-Vertigo:self	0.014462	-0.57868	0.607606	1
Vertigo:pollinated-Vertigo:self	0.268147	-0.28841	0.824703	0.875482
Hedin:pollinated-Vertigo:self	0.207928	-0.34397	0.759826	0.971405
Diana:pollinated-Hedin:self	-0.47595	-1.01984	0.067949	0.144303
Fuego:pollinated-Hedin:self	0.312574	-0.23718	0.862333	0.725122
Fury:pollinated-Hedin:self	-0.10937	-0.6857	0.466958	0.999847
Vertigo:pollinated-Hedin:self	0.144316	-0.39428	0.682914	0.997539
Hedin:pollinated-Hedin:self	0.084097	-0.44969	0.617881	0.999968
Fuego:pollinated-Diana:pollinated	0.788523	0.314828	1.262217	1.04E-05
Fury:pollinated-Diana:pollinated	0.366579	-0.13771	0.870865	0.379393
Vertigo:pollinated-Diana:pollinated	0.620265	0.159571	1.080959	0.001012
Hedin:pollinated-Diana:pollinated	0.560046	0.104989	1.015102	0.004283
Fury:pollinated-Fuego:pollinated	-0.42194	-0.93255	0.08866	0.205686
Vertigo:pollinated-Fuego:pollinated	-0.16826	-0.63586	0.299342	0.978959
Hedin:pollinated-Fuego:pollinated	-0.22848	-0.69052	0.23357	0.857334
Vertigo:pollinated-Fury:pollinated	0.253686	-0.24488	0.752252	0.835111
Hedin:pollinated-Fury:pollinated	0.193466	-0.2999	0.686828	0.962917
Hedin:pollinated-Vertigo:pollinated	-0.06022	-0.50893	0.38849	0.999992

Table S6: Tukey multiple comparisons of means for cage experiment, pod number (95% family-wise confidence level)

Fit: aov(formula = log(podnum + 1) ~ cv + poll2 + year + cv:poll2 + year:poll2, data = data3)

Statistical significance of tested model parameters
poll:cv:year (F= 1.066, p=0.374)
poll:cv (F=2.686, p=0.032)
poll:year (F= 1.154, p=0.284)
cv:year (F=0.981, p=0.419)
year (F=9.822, p=0.002)

$cv	diff	lwr	upr	p
Fuego-Diana	0.084926	-0.1613	0.331151	0.877833
Fury-Diana	0.339146	0.069715	0.608576	0.00571
Vertigo-Diana	0.372929	0.131062	0.614796	0.000306
Hedin-Diana	0.668734	0.430718	0.906749	0
Fury-Fuego	0.25422	-0.01521	0.523651	0.074778
Vertigo-Fuego	0.288003	0.046136	0.529871	0.01061
Hedin-Fuego	0.583808	0.345793	0.821824	0
Vertigo-Fury	0.033783	-0.23167	0.299237	0.996776
Hedin-Fury	0.329588	0.067639	0.591537	0.005738
Hedin-Vertigo	0.295805	0.062301	0.529309	0.005294

$poll2	diff	lwr	upr	p
pollinated-self	0.110765	-0.00841	0.229944	0.068374

$year	diff	lwr	upr	p
2018cage-2017cage	-0.86277	-0.97503	-0.75051	0

$\`cv:poll2\`	diff	lwr	upr	p
Fuego:self-Diana:self	0.359266	-0.13239	0.850921	0.371639
Fury:self-Diana:self	0.774564	0.204703	1.344424	0.000856
Vertigo:self-Diana:self	0.748292	0.256637	1.239947	8.99E-05
Hedin:self-Diana:self	1.402139	0.921667	1.882611	0
Diana:pollinated-Diana:self	0.659489	0.226105	1.092873	9.02E-05
Fuego:pollinated-Diana:self	0.6299	0.192447	1.067353	0.000288
Fury:pollinated-Diana:self	0.792208	0.336244	1.248172	3.3E-06
Vertigo:pollinated-Diana:self	0.85718	0.42747	1.28689	0
Hedin:pollinated-Diana:self	0.966827	0.54045	1.393204	0
Fury:self-Fuego:self	0.415298	-0.14266	0.973251	0.34465
Vertigo:self-Fuego:self	0.389026	-0.08878	0.866828	0.22348
Hedin:self-Fuego:self	1.042873	0.576585	1.509161	0
Diana:pollinated-Fuego:self	0.300223	-0.11738	0.717826	0.395838
Fuego:pollinated-Fuego:self	0.270634	-0.15119	0.692459	0.565805
Fury:pollinated-Fuego:self	0.432942	-0.00805	0.873934	0.05913
Vertigo:pollinated-Fuego:self	0.497914	0.084124	0.911703	0.005914
Hedin:pollinated-Fuego:self	0.607561	0.197235	1.017888	0.000163
Vertigo:self-Fury:self	-0.02627	-0.58423	0.531681	1
Hedin:self-Fury:self	0.627575	0.07945	1.1757	0.011446
Diana:pollinated-Fury:self	-0.11507	-0.62243	0.39228	0.999339
Fuego:pollinated-Fury:self	-0.14466	-0.6555	0.366172	0.996244
Fury:pollinated-Fury:self	0.017644	-0.50913	0.544418	1
Vertigo:pollinated-Fury:self	0.082616	-0.4216	0.586836	0.999956
Hedin:pollinated-Fury:self	0.192263	-0.30912	0.693646	0.96789
Hedin:self-Vertigo:self	0.653847	0.187559	1.120135	0.000483
Diana:pollinated-Vertigo:self	-0.0888	-0.50641	0.328801	0.999609
Fuego:pollinated-Vertigo:self	-0.11839	-0.54022	0.303433	0.99649
Fury:pollinated-Vertigo:self	0.043916	-0.39708	0.484908	0.999999
Vertigo:pollinated-Vertigo:self	0.108888	-0.3049	0.522677	0.997859
Hedin:pollinated-Vertigo:self	0.218536	-0.19179	0.628862	0.794463
Diana:pollinated-Hedin:self	-0.74265	-1.14703	-0.33827	6E-07
Fuego:pollinated-Hedin:self	-0.77224	-1.18097	-0.3635	3E-07
Fury:pollinated-Hedin:self	-0.60993	-1.03842	-0.18144	0.000362
Vertigo:pollinated-Hedin:self	-0.54496	-0.9454	-0.14452	0.000837
Hedin:pollinated-Hedin:self	-0.43531	-0.83217	-0.03845	0.019173
Fuego:pollinated-Diana:pollinated	-0.02959	-0.38177	0.322594	1
Fury:pollinated-Diana:pollinated	0.132719	-0.24221	0.507647	0.981172
Vertigo:pollinated-Diana:pollinated	0.197691	-0.14483	0.540209	0.707317
Hedin:pollinated-Diana:pollinated	0.307338	-0.03099	0.645665	0.111017
Fury:pollinated-Fuego:pollinated	0.162308	-0.21732	0.541932	0.93662
Vertigo:pollinated-Fuego:pollinated	0.22728	-0.12037	0.574932	0.538338
Hedin:pollinated-Fuego:pollinated	0.336927	-0.0066	0.680451	0.059641
Vertigo:pollinated-Fury:pollinated	0.064972	-0.3057	0.435647	0.999921
Hedin:pollinated-Fury:pollinated	0.174619	-0.19219	0.541425	0.88319
Hedin:pollinated-Vertigo:pollinated	0.109647	-0.22396	0.443255	0.988779

$`poll2:year`	diff	lwr	upr	p
pollinated:2017cage-self:2017cage	0.309891	0.091957	0.527825	0.001632
self:2018cage-self:2017cage	-0.63287	-0.88912	-0.37663	0
pollinated:2018cage-self:2017cage	-0.6681	-0.88475	-0.45145	0
self:2018cage-pollinated:2017cage	-0.94277	-1.16919	-0.71634	0
pollinated:2018cage-pollinated:2017cage	-0.97799	-1.15839	-0.79759	0
pollinated:2018cage-self:2018cage	-0.03522	-0.26042	0.189967	0.977573

Table S7: Tukey multiple comparisons of means for cage experiment, dominant node (95% family-wise confidence level)

Fit: aov(formula = maxnode.pod ~ poll2 * cv + year, data = data3a)

$poll2	diff	lwr	upr	p
pollinated-self	-2.16936	-3.22704	-1.11167	7.12E-05

$cv	diff	lwr	upr	p
Fuego-Diana	-1.2779	-3.46307	0.907268	0.494373
Fury-Diana	-2.9329	-5.324	-0.54179	0.007685
Vertigo-Diana	-1.0905	-3.23699	1.055991	0.630847
Hedin-Diana	-6.12437	-8.23668	-4.01207	0
Fury-Fuego	-1.65499	-4.0461	0.736111	0.319028
Vertigo-Fuego	0.187402	-1.95909	2.333891	0.999264
Hedin-Fuego	-4.84647	-6.95878	-2.73417	0
Vertigo-Fury	1.842397	-0.51341	4.198207	0.202941
Hedin-Fury	-3.19148	-5.51619	-0.86677	0.001874
Hedin-Vertigo	-5.03388	-7.10615	-2.96161	0

$year	diff	lwr	upr	p
2018cage-2017cage	-1.01239	-2.00866	-0.01612	0.046433

$`poll2:cv`	diff	lwr	upr	p
pollinated:Diana-self:Diana	-6.88678	-10.7329	-3.04067	1.4E-06
self:Fuego-self:Diana	-5.47833	-9.84157	-1.11509	0.003176
pollinated:Fuego-self:Diana	-6.09488	-9.9771	-2.21266	4.43E-05
self:Fury-self:Diana	-6.54363	-11.6009	-1.48635	0.001979
pollinated:Fury-self:Diana	-8.14095	-12.1874	-4.09445	0
self:Vertigo-self:Diana	-5.24442	-9.60766	-0.88117	0.006006
pollinated:Vertigo-self:Diana	-5.98933	-9.80284	-2.17582	4.39E-05
self:Hedin-self:Diana	-9.86921	-14.1332	-5.60521	0
pollinated:Hedin-self:Diana	-11.2142	-14.9982	-7.43031	0
self:Fuego-pollinated:Diana	1.408452	-2.29761	5.114514	0.969726
pollinated:Fuego-pollinated:Diana	0.791903	-2.33358	3.91739	0.998407
self:Fury-pollinated:Diana	0.343148	-4.15942	4.84572	1
pollinated:Fury-pollinated:Diana	-1.25417	-4.5815	2.073167	0.971316
self:Vertigo-pollinated:Diana	1.642366	-2.0637	5.348427	0.921746
pollinated:Vertigo-pollinated:Diana	0.897453	-2.14226	3.937163	0.994866
self:Hedin-pollinated:Diana	-2.98242	-6.57111	0.606265	0.199074
pollinated:Hedin-pollinated:Diana	-4.32745	-7.32997	-1.32494	0.000282
pollinated:Fuego-self:Fuego	-0.61655	-4.36008	3.126978	0.999954
self:Fury-self:Fuego	-1.0653	-6.01692	3.88631	0.99957
pollinated:Fury-self:Fuego	-2.66262	-6.57625	1.251009	0.478888
self:Vertigo-self:Fuego	0.233913	-4.0064	4.474223	1
pollinated:Vertigo-self:Fuego	-0.511	-4.18322	3.161216	0.999989
self:Hedin-self:Fuego	-4.39088	-8.529	-0.25276	0.027681
pollinated:Hedin-self:Fuego	-5.7359	-9.37739	-2.09442	0.000041
self:Fury-pollinated:Fuego	-0.44876	-4.98222	4.084704	0.999999
pollinated:Fury-pollinated:Fuego	-2.04607	-5.41509	1.322944	0.642511
self:Vertigo-pollinated:Fuego	0.850462	-2.89307	4.59399	0.999331
pollinated:Vertigo-pollinated:Fuego	0.10555	-2.97973	3.190828	1
self:Hedin-pollinated:Fuego	-3.77433	-7.4017	-0.14696	0.033951
pollinated:Hedin-pollinated:Fuego	-5.11936	-8.16799	-2.07072	8.3E-06
pollinated:Fury-self:Fury	-1.59732	-6.27222	3.077591	0.985259
self:Vertigo-self:Fury	1.299218	-3.6524	6.250832	0.997908
pollinated:Vertigo-self:Fury	0.554305	-3.92045	5.02906	0.999996
self:Hedin-self:Fury	-3.32557	-8.18996	1.538818	0.471574
pollinated:Hedin-self:Fury	-4.6706	-9.12017	-0.22103	0.031019
self:Vertigo-pollinated:Fury	2.896533	-1.0171	6.810161	0.352935
pollinated:Vertigo-pollinated:Fury	2.15162	-1.13797	5.441215	0.537677
self:Hedin-pollinated:Fury	-1.72826	-5.53093	2.074411	0.909586
pollinated:Hedin-pollinated:Fury	-3.07328	-6.32854	0.181969	0.08244
pollinated:Vertigo-self:Vertigo	-0.74491	-4.41713	2.927303	0.999734
self:Hedin-self:Vertigo	-4.62479	-8.76291	-0.48667	0.015397
pollinated:Hedin-self:Vertigo	-5.96982	-9.6113	-2.32833	1.54E-05
self:Hedin-pollinated:Vertigo	-3.87988	-7.4336	-0.32615	0.020222
pollinated:Hedin-pollinated:Vertigo	-5.2249	-8.18554	-2.26427	2.1E-06
pollinated:Hedin-self:Hedin	-1.34503	-4.86699	2.176934	0.968747

Table S8: - Tukey multiple comparisons of means for field experiment, bean number (95% family-wise confidence level)

Fit: aov(formula = bean.number ~ variety, data = data9)

Statistical significance of tested model parameters
poll:cv (F=1.027, p=0.395)
poll (F=0.098, p=0.907)
cv (F=3.43, p=0.034)

$variety	diff	lwr	upr	p
fury-fuego	4.244914	0.378081	8.111747	0.027518
vertigo-fuego	1.802817	-2.07751	5.683147	0.517209
vertigo-fury	-2.4421	-6.30893	1.424736	0.297432

Table S9: - Tukey multiple comparisons of means for field experiment, pod number (95% family-wise confidence level)

Fit: aov(formula = podnum ~ variety, data = data9)

Statistical significance of tested model parameters
poll:cv (F=1.05, p=0.4)
poll (F=0.007, p=0.99)
cv (F=7.5, p<0.001)

$variety	diff	lwr	upr	p
fury-fuego	2.352113	0.793982	3.910244	0.001312
vertigo-fuego	0.425861	-1.12685	1.978572	0.794055
vertigo-fury	-1.92625	-3.47896	-0.37354	0.010528

Table S10: Linear regression model summary for log-log plots for cage experiment. Note that variable beannulog is log(bean number + 1)

Fit: lm(formula = log(beanmass + 1) ~ beannulog + poll + cv + poll:cv + poll:beannulog + poll:year + cv:beannulog + cv:year + beannulog:year + poll:cv:beannulog + cv:year:beannulog, data = data3)

Coefficients	Estimate	Std. Error	t value	p
(Intercept)	-0.71074	0.20904	-3.4	0.000803
beannulog	0.87823	0.0892	9.845	<0.001
polltrip	-0.35197	0.24321	-1.447	0.149293
pollhand	-0.36956	0.25911	-1.426	0.155241
cvFuego	-0.23165	0.36794	-0.63	0.529632
cvFury	0.63062	0.26465	2.383	0.018052
cvVertigo	0.56155	0.31159	1.802	0.072912
cvHedin	0.514	0.73502	0.699	0.485115
polltrip:cvFuego	-0.18157	0.30793	-0.59	0.556042
pollhand:cvFuego	0.21097	0.3479	0.606	0.544872
polltrip:cvFury	0.36946	0.31203	1.184	0.237703
pollhand:cvFury	-0.69942	0.35154	-1.99	0.047904
polltrip:cvVertigo	-0.11128	0.34063	-0.327	0.74423
pollhand:cvVertigo	-0.83214	0.39542	-2.104	0.036501
polltrip:cvHedin	0.20912	0.45066	0.464	0.643092
pollhand:cvHedin	0.01167	0.4571	0.026	0.979654
beannulog:polltrip	0.05569	0.0911	0.611	0.541667
beannulog:pollhand	0.0094	0.09946	0.095	0.924793
pollself:year2018cage	0.46214	0.20403	2.265	0.024508
polltrip:year2018cage	0.55321	0.23847	2.32	0.021289
pollhand:year2018cage	0.59472	0.22079	2.694	0.007625
beannulog:cvFuego	0.22078	0.13176	1.676	0.095262
beannulog:cvFury	-0.1108	0.10398	-1.066	0.287836
beannulog:cvVertigo	-0.05651	0.11254	-0.502	0.61607
beannulog:cvHedin	-0.17336	0.19128	-0.906	0.36577
cvFuego:year2018cage	-0.21274	0.36932	-0.576	0.565191
cvFury:year2018cage	-0.8339	0.31239	-2.669	0.008177
cvVertigo:year2018cage	-0.24557	0.34195	-0.718	0.473444
cvHedin:year2018cage	-1.29271	0.72972	-1.772	0.077892
beannulog:year2018cage	-0.18063	0.08298	-2.177	0.030582
beannulog:polltrip:cvFuego	0.0304	0.1166	0.261	0.794568
beannulog:pollhand:cvFuego	-0.0821	0.12677	-0.648	0.517901
beannulog:polltrip:cvFury	-0.07131	0.11392	-0.626	0.532024
beannulog:pollhand:cvFury	0.29211	0.12743	2.292	0.022857
beannulog:polltrip:cvVertigo	0.07687	0.11747	0.654	0.513597
beannulog:pollhand:cvVertigo	0.30956	0.13625	2.272	0.024076
beannulog:polltrip:cvHedin	-0.01944	0.13367	-0.145	0.884519
beannulog:pollhand:cvHedin	0.06803	0.14128	0.482	0.630625
beannulog:cvFuego:year2018cage	0.20025	0.12391	1.616	0.107538
beannulog:cvFury:year2018cage	0.3404	0.11057	3.079	0.00235
beannulog:cvVertigo:year2018cage	0.14302	0.11569	1.236	0.217722
beannulog:cvHedin:year2018cage	0.4458	0.19701	2.263	0.024648

Residual standard error: 0.1943 on 215 degrees of freedom
(5 observations deleted due to missingness)
Multiple R-squared:0.955; Adjusted R-squared: 0.9464; F-statistic: 111.3 on 41 and 215 DF; p-value: < 2.2e-16

Table S11: Linear regression model summary for log-log plots for field experiment. Note that variable beannulog is log(bean number + 1)

Fit: lm(formula = log(bean.mass + 1) ~ beannulog + treatment, data = data9)

Coefficients	Estimate	Std. Error	t value	p
(Intercept)	-0.36163	0.059592	-6.068	5.99E-09
beannulog	0.85249	0.019951	42.728	< 2e-16
polltrip	0.00765	0.032698	0.233	0.8163
pollopen	-0.07631	0.032789	-2.327	0.0209

Residual standard error: 0.1946 on 209 degrees of freedom
(3 observations deleted due to missingness)
Multiple R-squared: 0.8974; Adjusted R-squared: 0.896;F-statistic: 609.6 on 3 and 209 DF; p-value: < 2.2e-16
